# A qualitative study of the views of adolescents on their caries risk and prevention behaviours

**DOI:** 10.1186/s12903-015-0128-1

**Published:** 2015-11-10

**Authors:** Emma Hall-Scullin, Joanna Goldthorpe, Keith Milsom, Martin Tickle

**Affiliations:** School of Dentistry, University of Manchester, Oxford Road, Manchester, M13 9PL England; University of Manchester, Dental Health Unit, Williams House, Manchester Science Park, Lloyd Street North, Manchester, M15 6SE England; Department of Dental Public Health, 1829 Building, Countess of Chester Hospital, Chester, England

**Keywords:** Dental caries, Oral health, Adolescence, Qualitative, Semi-structured interviews, Influences, Behaviour, Knowledge, Attitudes

## Abstract

**Background:**

The purpose of this study was to explore the attitudes and beliefs of adolescents towards dental caries and their use or non-use of caries prevention regimens.

**Methods:**

Adolescents aged 16 years from four state-funded secondary schools in North West of England (*n* = 19). Purposive sampling strategically selected participants with characteristics to inform the study aims (gender, ethnicity, and caries status). Semi-structured interviews were transcribed verbatim and analysed using a framework approach.

**Results:**

14 codes within five overarching themes were identified: “Personal definition and understanding of oral health”; “Knowledge of oral health determinants”; “Influences on oral health care”; Reason for oral health behaviours”; and “Oral health in the future”. Adolescents conceptualise oral health as the absence of oral pathology and the ability to function, which included an aesthetic component. Appearing to have healthy teeth was socially desirable and equated with positive self-image. The dominant influence over oral health behaviours was habitual practice encouraged by parents from a young age, with limited reinforcement at school or by dental practices. At this transitional age, participants recognised the increasing influence of peers over health behaviours. Self-efficacy pertained to diet modification (reduction in sugar-ingestion) and oral hygiene behaviour (tooth-brushing). A lack of understanding of caries aetiology was evident. Behaviours were mitigated by a lack of environmental support; and a desire for immediate gratification often overcame attempts at risk-reducing behaviour.

**Conclusions:**

Parents primarily influence the habitual behaviours of adolescents. With age, the external environment (availability of sugar and peers) has an increasing influence on behaviour. This suggests that to improve adolescent health, oral health promoters should engage with parents from early childhood and create supportive environments including public policy on sugar availability to encourage uptake of risk-minimising behaviours.

## Background

The 2013 UK Children’s Dental Health Survey found that obvious decay experience (D_3_MFT > 0) in the permanent teeth was evident in 34 % of 12-year-olds and 46 % of 15-year-olds [1]. Some 58 % of children aged 12 and 45 % of those aged 15 reported that their daily life had been affected by problems with their teeth and mouth in the last three months [2]. The 2002/03 Regional Dental Health Survey reported that 57 % of 14-year-olds in the North West of England had previous caries experience with active decay present in 33 % [3]. Caries is a largely preventable disease but uptake of preventive practices are variable and affected by multidimensional aetiological factors acting at the psycho-social and environmental level [4].

Adolescence represents a period of transition when children are establishing autonomy over their own behaviours [5]. Social influences become increasingly important with an expectation that individuals will adopt the behaviours accepted as the social norm within their peer group, for example, with respect to smoking, eating the same foods, and other behaviours that can impact on oral health [6–8]. However the personal views of adolescents about such influences have not been extensively explored.

The majority of studies exploring caries in adolescents are quantitative [1, 2]. These studies provide useful descriptions of population trends in caries or uptake of preventive practice but not for exploring the reasons behind an individual’s actions [9]. When a phenomenon is only partly understood, a qualitative design is preferable [10]. However, few studies have used qualitative methodology to investigate oral health in adolescents, particularly within the United Kingdom [8, 11].

A number of previous qualitative studies exploring the oral health influences and behaviours in adolescents used focus groups. However, adolescents can be strongly influenced by peer pressure and focus groups comprised of groups of children from the same peer group are noted to produce more critical comments about the effectiveness of oral health preventive practices [12]. Other qualitative studies focused only on participants with specific characteristics e.g. pregnancy and orthodontic appliances that further limited the findings applicability to the wider population [13–15]. Few studies exploring oral health were also able to describe the oral health of the participants and it is not clear how previous caries experience impacted on reported behaviours and attitudes.

The aim of this study was to explore the attitudes and beliefs of adolescents towards dental caries and their use or non-use of caries prevention regimens when caries status was known. It allowed adolescents views to be explored in an environment where they would not be directly influenced by their peers, in addition to providing a link to oral health -related characteristics of the participants.

## Methods

### Subjects

Participants were recruited through state-funded secondary schools (attended by students aged 11–16 years) in the North West of England. The inclusion criterion for schools was participation in the outcome component of the East Lancashire and Manchester Study (ELMS) (NIHR UK Clinical Research Network Study Portfolio, Ref: 10315). The study was a whole population prospective cohort study of children conducted in state-funded schools in the North West of England with data collected at four time points over seven years. Dental assessments were carried out at four time points supplemented at the last two time points with questionnaires about self-reported oral health behaviours and height and weight measures.

All students registered in secondary schools that took part in ELMS, who agreed to take part in the study and had the ability to consent, were eligible to participate. Subjects were excluded if they were not able to converse in English or unable to give consent. Six schools agreed to provide a list of potential participants. Caries status plus socio-demographic data was available for all participants from the ELMS dataset. The school provided individual level date of birth, gender, and home postcode data. A postcode is assigned to a geographical area usually comprising up to 25 houses. Postcodes were used to estimate small-area measure of socioeconomic status to participants based on national quintiles of the Lower Super Output Area (LSOA) Index of Material Deprivation 2010 (IMDQ, 2010) [16, 17]. IMD Quintile 1 (IMDQ 1) was the least deprived and IMD Quintile 5 (IMDQ 5) the most deprived. For the purpose of this study, participants were recoded as IMDQ1-3 (less deprived) IMDQ 4–5 (more deprived). Ethnicity was reported by the parent according to nationally agreed categories and coded as white, Asian, black, Chinese, mixed or other [18].

Purposive sampling was used to identify subjects with characteristics of relevance to the aim of the study and maximise variation within the sample. Criteria included caries status, gender and socio-economic status (Table 1). This non-probability approach is suitable for in-depth qualitative research in which the focus is to understand complex social phenomena [19]. Planned number for recruitment was aligned with previous research [8, 20–23] but actual recruitment was determined by theme saturation i.e. when no new information was generated from the analyses, no further interviews were conducted [24].

**Table 1:** Sampling framework and characteristics of participants recruited

		Gender number (actual recruited)				Total
		Male		Female		
		IMDQ category		IMDQ category		
		IMDQ 1-3	IMDQ 1-3	IMDQ 1-3	IMDQ 4-5	
Caries status	Caries free	3 (0)	3 (2)	3 (1)	3 (1)	12 (4)
	Caries	6 (1)	6 (5)	6 (4)	6 (5)	24 (15)
	Total	9 (1)	9 (7)	9 (5)	9 (6)	36 (19)

All parents and potential participants were informed of the study by participant information sheet (*n* = 41) and advised of how to opt-out. Adolescents who did not withdraw at this stage were invited to participate. Refusals were received from 18 potential participants and four failed to attend for interview. Formal written consent was obtained from participants. Theme saturation was reached when a total of 19 participants were recruited. The University of Manchester Ethics Committee granted ethical approval (Ref: 14106).

### Procedure

The study was conducted in schools during June-July 2014. Interviews were conducted in small rooms at each participant’s school. The interviewer (EHS) attended sessions in collecting qualitative interview data provided by Academic and Researcher Development, University of Manchester. This included multi-disciplinary interactive sessions. A literature search was conducted to inform the theoretical framework within which our research questions lay; the development of the topic guide; and the range and depth of perspectives sought. [8, 20, 25–30] Discussions were recorded digitally and transcribed verbatim. The length of interview was not fixed and determined in the field by the interviewer using participants’ verbal and non-verbal cues to revise the time. After each interview, field records were completed to capture the interviewer’s reflections on the process including non-verbal cues; the success or failure; information given after the interview and consideration of future modifications.

### Interview schedule

A. Perception
  Tell me about what you think is a healthy mouth?
  What do you think is meant by “tooth decay”?
  Why do you think teenagers get tooth decay?
  Why do you look after your teeth?
  Tell me about people who could help you look after your teeth?
B. Behaviour
  What do you do to prevent tooth decay?
  What do you do to take care of your healthy teeth?
  What do you do to take care of tooth decay?
  What is the role of diet in keeping your teeth healthy?
  Who influences why you look after your teeth?
  Where would you go for advice or information about looking after your teeth?

### Data analysis

Transcripts and field records were uploaded into NVivo 10 software that supports the Framework approach that was used in analysis [31]. This method was chosen because it supported exploration of specific a priori themes identified from the literature search and analyses of the outcomes of the preceding quantitative study; and facilitated an inductive approach to facilitate the emergence of new themes. A grounded theory (reference) approach was also considered, however we decided to pragmatically explore specific issues of interest, in addition to generating new, user generated themes. Grounded theory would have been more appropriate for the generation of a new, encompassing theory of oral health [32].

After coding, the data was analysed by case and theme to identify variables (gender, previous caries status and socio-demographic status) associated with specific explanatory models, typologies or themes.

Two researchers (EHS and JG) analysed the transcripts. Codes were assigned to categories and sub-categories relating to the patterns or themes that emerged. This enabled questions to be refined and new avenues of inquiry to develop based on patterns of responses. Analysis produced a coding frame with 14 codes arranged within five overarching themes each with a description and example to ensure consistency of coding. When theme saturation was reached i.e. when no further themes were generated from the analyses, no further interviews were conducted [24]. This was the point at which the collection of new data was deemed not to shed new light in the area under investigation.

This final analytical framework was applied to each transcript systematically. Interpretation was based on the original research objectives and new concepts generated inductively from the data. Agreement between researchers was reached by discussion and review of the transcripts.

The framework analysis produced a coding frame with 14 codes organised into five overarching themes. This paper presents the first two themes relating to participant’s definition and knowledge of oral health with an emphasis on caries. The third thematic discussion relates to the participants’ perception of influences (social, professional and external) on their oral health behaviours and attitudes. This focused primarily on the social support of their parents. The paper concludes with the reasons for oral health behaviour (habitual behaviour, delayed uptake and lifestyle choice) that participants expressed and how participants perceive their health behaviours will change in the future.

### Reflexivity

The two data analysts were a health services researcher with a background in health psychology (JG) and a Specialty Registrar in Dental Public Health with a background in Paediatric and Special Care Dentistry (EHS). They reflected on their own assumptions before starting to identify potential sources of bias. JG had previous experience of analysing qualitative dental data and believed that there would be a gender difference in reported behaviours with males demonstrating less frequent oral hygiene behaviours. EHS had no previous experience of conducting qualitative research but received training. EHS’s view was that adolescents primarily viewed health as something that could be seen and that individuals were responsible for their own behaviours.

### Quality

The semi-structured interview guide provided a clear set of instructions for the interviewer and a degree of comparison by standardising at least some of the questions. A verbal “debrief” conducted after every session with a member of the research team supported the reflective process. An audit trail of the process, field notes, memos and summaries of the data evidenced transparency of the process.

The transferability of semi-structured interviews depends on whether participants’ opinions are truly reflected and it cannot be guaranteed that participants will tell the truth. Threats to validity include the use of leading questions; the interviewer’s preconceived ideas influencing the discussion; and the participants’ perception of what the interviewer wants to hear. It was impossible to fully evaluate these effects but it was feasible to examine the internal consistency of what the participant said.

Data was transcribed verbatim and accuracy checked by reading the transcript while listening to the audio files. Two trained analysts conducted analysis as soon as possible after transcription [33]. Adopting a participatory approach in which transcriptions are co-created and evaluated increased credibility. Credibility (internal validity) was assessed by prolonged and persistent observation until thematic saturation was reached. Commonalities and differences were identified before relationships based on participants’ characteristics and thematic similarities were considered.

Conclusions were both descriptive and explanatory. Deviant cases (outliers) were included in analysis. Comparison of outcomes was conducted and discussed to reach consensus, not about the codes applied but the reason for dissonance to reduce biased reporting. The output did not deliver a single definitive explanation for oral health attitudes and behaviours but generated a model for future research. The dependability (reliability) and consistency of data was considered by triangulation. Confirmatory evidence (objectivity) was sought by comparison across the transcripts, field notes and with the quantitative data (transferability). In future, member checks would be recommended (respondent validity). This was not logistically possible here as participants left their schools shortly after the research was conducted. The implications for policy i.e. transferability are discussed in the results.

## Results

The mean age of participants was 16.2 years (SD = 0.30) with 8 (42.1 %) male. Participants were from the white (11, 57.9 %) and Asian communities (8, 42.1 %). Caries was experienced by 78.9 % (*n* = 14). The mean D_3_MFT caries in those with the disease was 3.07 (IQR 1–4). Saturation of themes was reached when 19 students had been interviewed and no further recruitment was conducted (Tables 1, 2, 3) [24].

**Table 2:** Recruitment frequency

Schools (*n* =24)	
Refused n	11
Agreed n	6
Recruited from n	4
Subjects (*n* = 41)	
Refused n	18
Failed to attend n	4
Recruited (*n* = 19)	
Male n (%)	8 (42.1)
Age years (SD)	16.2 (0.30)
White n (%)	11 (57.9)
IMDQ (2010) Mode (%)	5 (52.6)
DMFT = 0 n (%)	4 (21.1)
DMFT > 0 Mean (IQR)	3.07 (1.00 – 4.00)

**Table 3:** Individual participant characteristics

ID Number	D_3_MFT^a^	Gender	IMDQ (2010)^b^
1	2	F	IMDQ 1-3
2	1	F	IMDQ 4-5
3	0	M	IMDQ 4-5
4	0	F	IMDQ 1-3
5	1	M	IMDQ 1-3
6	1	F	IMDQ 1-3
7	0	F	IMDQ 4-5
8	1	F	IMDQ 1-3
9	0	M	IMDQ 4-5
10	2	F	IMDQ 4-5
11	2	F	IMDQ 4-5
12*	4	M	IMDQ 4-5
13	1	F	IMDQ 4-5
14*	3	M	IMDQ 4-5
15	2	M	IMDQ 4-5
16	2	M	IMDQ 4-5
17*	9	M	IMDQ 4-5
18	5	F	IMDQ 4-5
19*	10	F	IMDQ 4-5

*D_3_MFT > 2 ^a^Caries status is presented using the D_3_MFT Index (Decayed, Missing, Filled Teeth Index), which is a summary value of the total number of teeth affected by caries into dentine in an individual. ^b^Participant’s home postcodes were used to generate estimates of individual level measure of deprivation based on their Lower Super Output Area of the national Index of Material Deprivation Quintiles 2010 (IMDQ)

### Personal definition and understanding of oral health

Oral health was defined as being free from disease and aesthetically pleasing.

*Participant 8 (Female, caries): White teeth, not sore gums, gums that don’t hurt when you brush your teeth…no yellow bits in between each tooth…*

Those who demonstrated the aesthetic ideal were seen as more socially acceptable and generally more worthy people.

*Participant 2 (Female, caries): …but if you’ve got nice teeth then it just shows that you look after yourself, you care about your teeth, you care about your hygiene, you’ve learnt, so it kind of shows that you come from a good background as well.*

The participants suggested a link between general and oral health. Improving general health would improve oral health and vice versa. Both were equally important.

*Participant 11 (Female, caries): Yeah, because you take in everything from your mouth. So if you’re having too much fat, it’s going in through your mouth, so at first it’ll affect your teeth, and then after going in it’ll affect your body.*

### Knowledge of oral health determinants

#### Diet modification

Individuals appeared to know about diet modification but in-depth exploration revealed clear misunderstandings in relation to dental caries. Fat and sugar ingestion were considered as causal agents of dental caries. Participants were unsure how quickly following exposure caries developed and whether it was the frequency or amount of sugar consumed that was worse.

*Participant 1 (Female, caries): Sprite, or something, which might contain less sugar, you can have them more often but not compared to the other unhealthy drinks.*

#### Oral hygiene habits

The main oral hygiene habit practiced was tooth-brushing which interviewees believed could mitigate the impact of eating sugar.

*Participant 3 (Male, caries-free): If you eat too much of the sugary foods then it’s harmful to your teeth, but if you eat a few during the day, but then you brush your teeth afterwards, then it will be fine.*

The roles of fluoride or plaque in dental caries were not understood.

*Participant 7 (Female, caries-free): I don’t know, fluoride sounds like the type of thing that it might gently whiten them. I imagine it strengthens the teeth, but I’m not really sure how it works.*

Mouthwash was used as alternative to tooth brushing because the media presented it as an effective prevention.

Participant 12* (Male, caries): It depends. Like, if I wake up late and I can’t be bothered brushing my teeth. So I just use a mouthwash and then that’s it.

### Influences on oral health care

#### *Social support-* Family

The dominant influence on participants’ personal attitudes and beliefs for oral health was family. Participants thought dental caries was attributable to lack of parental involvement. There were no differences due to gender, ethnicity or caries status.

*Participant 7 (Female, caries-free): …there is enough, at the minute, advice, through adverts, and on the Internet as well, which tells you about your dental care. So, it’s parents that don’t acknowledge it and pass it on to their children, which don’t particularly move along with the information to keep it themselves.*

#### Social support- Peers

Participants expected their peers to practice those behaviours associated with the social norm even if they did not personally. There was a reported similarity of behaviours within a group. Unhealthy activities were perceived negatively.

*Participant 5 (Female, caries): Well my group of friends because they have similar interests in what I do. It’s like because I’m quite sporty I’ve like the football team, we’re all quite good mates and stuff. So I’ve quite…I’ve stuck to them and they don’t go and do all the stupid things like smoking and stuff, so.*

#### Professional influences- Dentist

The dentist was seen as supporting oral health behaviours advocated by parents.

*Participant 8 (Female, caries) My mum when I was little but I never really believed her, when I went to the dentist and it was like…I’ve actually got to do this…*

Preventive care by professionals was frequently mentioned but limited to advice and occasionally fluoride varnish. The same advice was purportedly given at every visit without tailoring or reviewing the outcome, which reduced the impact of the advice.

*Participant 6 (Female, caries): Yeah. I think he [the dentist] has like that little routine, like gives it to everyone. But I suppose you’ll get the message out, won’t you?*

*Participant 4 (Female, caries-free): They could maybe ask you certain lifestyle questions and then how often you brush your teeth, and then they could work out what you need to improve for it to not happen again.*

Using “real scientists and dentists” improved the credibility of adverts. Participants used these adverts to inform their oral health behaviours and to ask parents for particular products, especially mouthwash.

*Participant 5 (Female, caries): Yeah, if there’s like someone who like knows what they’re doing and like has understood it and probably like done a degree in it, say, you’re more likely to listen to them than someone who’s just like telling you, like, say, an actor or something that’s got a script saying, buy this product.*

#### Professional influences- School

School was considered the best place to deliver caries prevention advice to maximise the number of children reached. However, advice given to adolescents in school was uncommon and the impact on actual behaviours was unclear without parental support.

*Participant 1 (Female, caries): Not really the school’s responsibility, it’s more parent’s, but not everyone has got parents that will do it, so I think school would be a good idea to do it as well.*

*Participant 3 (Male, caries-free): It did tell us everything that they did to you, energy drinks, but no one really took heed of the advice, and they ignored it…*

It was perceived that the information lacked relevance with no penalties or rewards for engagement; and no change in the environment to support uptake.

*Participant 5 (Female, caries): … a bit of a doss* [easy, requiring little effort] *lesson. So they’re probably more likely to just sit there and chill rather than actually listen to what the message is saying, because it is important… you don’t get graded in PHSE* [Personal, Social and Health Education].

#### External influences

Participants acknowledged a number of celebrity role models whose habits and appearance they aspired to. Participants were aware that the media does manipulate images. They thought variation from this aesthetic was normal and not an indicator of poor health or the disease.

*Participant 2 (Female, caries): They’re trying to look good like the celebrities but act like the celebrities as well, but they don’t realise that it’s just like fake kind of…*

### Habitual behaviour

The dominant explanation for behaviour in adolescents was the promotion of habitual behaviours by parents from an early age. Reinforcing cues in adolescence included a desire to adopt the societal norm regarding health and appearance, particularly inspired by the images of celebrities.

*Participant 2 (Female, caries): You learn it from a young age don’t you, like your parents tell you to brush your teeth before you go to bed and when you wake up, and you just stick to the routine.*

*Participant 2 (Female, caries): Nobody wants horrible teeth do they? So you just brush them when you’re meant to. It’s not hard to brush your teeth.*

It was also suggested that habit was the reason participants failed to adopt certain behaviours. This was mitigated by a perceived lack of negative outcomes.

*Participant 1 (Female, caries): They might have got into a habit of not doing it, and not think there’s any point in doing it because they don’t see what it’s doing, if it’s inside.*

### Risk-related behaviour

Lack of uptake was associated with a lack of understanding of the immediacy of the effect of detrimental behaviours, especially sugar ingestion and an emphasis on instant self-gratification.

*Participant 7 (Female, caries-free): Like, my friend, she said, oh when I’m 18 I’ll just get veneers it doesn’t matter, and then she can carry on…like she just carries on eating the sweets…*

This delay related to addressing oral health with peers or accessing dental services unless there was overwhelming evidence of a “serious” effect.

*Participant 3 (Male, caries-free): …unless I knew it was a really bad problem, like it was affecting them…I don’t really think I’d talk to them unless it was that. I would talk to them, but I wouldn’t talk to them about their teeth!*

*Participant 3 (Male, caries-free): I don’t really think I would go as much as I should, but, because I know there’s nothing wrong with me at the minute…*

### Lifestyle choice

Participants acknowledged their personal autonomy and responsibility in adolescence to make choices about achieving and maintaining oral health. They suggested that adolescents with caries made a conscious lifestyle choice not to practice healthy behaviours.

*Participant 5 (Female, caries): Obviously you can be told to do things but without you actually doing them you won’t get anywhere, like your teeth won’t be nice or anything, so.*

Participant 14* (Male, caries): They just do everything, like, what’s not good for their teeth, like, they drink, they smoke to make themselves look cool, they eat all, like, sugar and when people say, are you not going to brush your teeth? They just act clever and say, no, I don’t have to when they really do…

### Oral health in the future

Currently, respondents were optimistic about their future oral health.

*Participant 8 (Female, caries): Pretty healthy, not the most healthiest in the world but pretty healthy. Hopefully no fillings till later on. Maybe a little bit yellow but mostly white.*

Although a few planned to get their teeth whitened.

*Participant 5 (Female, caries): Quite a few of my friends like say when they’re older they want their teeth whitening and stuff.*

Participants felt that looking after their teeth now was easy because they had the support of their parents, school and access to free dental care. As they moved on, they thought their oral health behaviours would change. It would be more difficult due to the cost of dental care (males particularly) and the greater accessibility of sugar rich foods.

*Participant 5 (Female, caries): Yeah, because we’re a healthy school. So, well, sixth form’s an exception, but they don’t serve any, in main school, they don’t serve anything like, with over a certain amount of sugar or a certain amount of salt, or they’re not allowed to serve fizzy drinks. So, it’s quite easy to be healthy at school, in main school. Sixth form, not quite as easy.*

*Participant 3 (Male, caries-free): Because, like, for adults, it costs money doesn’t it for adults? So people won’t do it because of the cost and they don’t want to spend the money.*

They would no longer have the support of their parents when attending the dentist, which frightened them, especially those who already had received treatment.

*Participant 11 (Female, caries): Taking someone with you. I think taking someone with you is better. Because I can’t think of going alone. I think even when I’m 50 I’ll still take my mum, I won’t go alone.*

## Discussion

This is the first study in the United Kingdom using semi-structured interviews to explore what adolescents think causes caries, and why they adopt certain approaches to caries prevention. The use of face-to-face interviews rather than focus groups gave valuable insight into the personal experiences and day-to-day behaviours of individuals [34]. The respondents represented a typical cross-section of the population in the area with high numbers of respondents from the white and Asian communities and higher representation from more deprived groups than the national averages [35]. Respondents presented with high levels of caries reflecting the high prevalence and severity in this region reported previously [36, 37].

It was anticipated that those with caries would report more dental problems and less uptake of caries preventive regimens [1, 2] but there was no apparent difference between those with and without caries. Nor did those with high caries report the feelings of inadequacy voiced by similar adolescents in Hattne [20]. This may be due to report bias or due to participants’ lack of perception about their actual behaviours. Theme saturation was reached before a high number of caries-free or affluent individuals were interviewed. Increasing the variation of participants may have revealed different patterns of behaviours. This could be addressed in future research.

There was also no evidence of the differences in behaviour related to gender reported previously [12, 14]. Whether this reflected report bias, genuine changes in gender norms or was due to previous participants’ conforming to expectations of behaviour in focus groups was unclear. Triangulation through discourse with other members of the research team, revisiting the transcript and comparing findings with other published studies helped to ensure that the findings had validity.

The dominant influence on participants’ caries preventive regimens was the habitual preventive behaviour reinforced by parents from early childhood. The link between parents and the oral health of children has been previously reported [20, 38]. This suggests that to improve adolescent health, oral health strategy should engage with parents [39–41]. A life-course approach from early childhood would encourage development of and support on-going practice. It was clear that during this transitional developmental period, parental influence was waning and external factors, including peer pressure, and popular media became increasingly relevant. Hedman [42] found that the desire to conform with peers was strong [14, 38], particularly in older adolescents as anticipated by Stokes [8]. The participant suggestion that “lifestyle” plays a role in behavioural choices suggests that, as in Trulsson [14], adolescents may not be fully conscious of the impact of external influences. This endorses the provision of supporting environments to encourage uptake of positive oral health behaviours [8].

This group perceived that the advice provide by professionals in school and at the dentist about oral health was inadequate and not tailored to the needs of the individual [22]. Despite the support for school-based delivery of oral health promotion [43] it seemed limited to the National Healthy Schools Programme (NHSP). As reported by Stokes [44], this programme uses a Common Risk Factor Approach (dietary modification) [45] to address a range of non-communicable diseases and does not specifically promote oral health behaviours such as tooth-brushing. Its purpose is to facilitate supportive environments in schools “making healthy choices easier” i.e. by restricting unhealthy foods in schools, children must choose healthier options. Evaluation of the NHSP has not demonstrated significant changes in pupil health-related behaviours following engagement with the NHSP [46]. Its focus on diet modification to address the overweight/obesity epidemic has also led to misunderstandings of the role of different food groups in health.

In-depth exploration identified significant knowledge gaps and misinterpretation similar to those identified by Ostberg [25]. There was marked confusion about what constituted a cariogenic or cariostatic diet. As in Murphey [13] and Hedman [42] participants underplayed the role of sugar ingestion, and were unclear of the role of tooth-brushing in prevention. There was no quality element to participants understanding of tooth-brushing beyond the requirement to perform it twice daily [42]. This lack of understanding had a negative impact on the efficacy of their oral health behaviours. Mouthwash was considered an effective alternative to tooth-brushing because participants did not understand the anti-cariogenic properties of fluoride toothpaste. Mouth cleaning (either with a tooth-brush or mouthwash) after ingesting sugar-rich food was understood to prevent caries; similarly to cleaning a wound would prevent infection by removing bacteria.

The participants had a holistic view of oral health and cited criteria from both the biomedical and psycho-social models [22]. This aligned with the views of adolescents in earlier studies, although previous participants gave greater primacy to aesthetics over oral health [20, 22]. Consideration was given to oral health as the absence of disease and freedom from pain, but they also acknowledged the social impact of an aesthetic smile [14]. Participants recognised that pleasing oral aesthetics did not equate with good oral health.

Previously, the attainment of general health was more valued than oral health [13]. Here, parity was given to both and attainment linked. This was related to participants’ superficial understanding of the role of diet in a multiplicity of illnesses including obesity and caries. Although all parties desired the aesthetic ideal, it was accepted that this did not always equate with health. This synergy could be utilised in future preventive strategy development. For example, an integrated health promotion strategy to improve oral and general health would tie-in with these participants’ holistic understanding of health. Promotion of body image could be used as a motivator [15]. Oral and general health were seen as equally important and closely interlinked.

In contrast to Ostberg, this group recognised that they could influence their own oral health [47]. This may reflect their holistic view of health beyond the bio-medical model. However, if participants practice behaviours they expect to be effective but still get caries long-term practice will be negatively impacted [20]. This has particular relevance as adolescents assume increasing responsibility for their oral health. It is important that they internalise cues for oral health behaviours to promote long-term effective self-care as parental influence wanes [48]. Those with more favourable dental health beliefs have better oral health when older [49].

Respondents were conscious of external influences beyond the education and health sectors. For example, Fitzgerald [22] found that participants used media adverts, especially as they grew older, to inform their knowledge about oral health [50]. In our study the veracity of information was improved by delivery by “dentists” or “scientists”. Unfortunately, this was evidenced by the erroneous use of mouthwash as an alternative to tooth brushing. This suggests that this age group does value the expertise and authority of professionals in the popular media, which could be utilised in the real world [20, 25].

In the future, participants expect that the loss of parental and environmental support as they leave school will coincide with a withdrawal from professional dental service and maintaining oral health will be more difficult [22]. Emphasising general health benefits could be used to motivate uptake as respondents highlighted the holistic model of health and the importance of aesthetics.

## Conclusions

Current professionally-led programmes of oral health prevention are inadequate to meet the needs of adolescents and efforts must be made to redress the deficiency [30]. National guidance relating to the delivery and commissioning of oral health prevention in childhood is based on limited evidence [30, 40] and the effectiveness of dental practice-based and school-based oral health education is unclear [51, 52]. This study demonstrated adolescents had persistent misunderstanding of caries causality and prevention, and this lack of understanding had a detrimental impact on their behaviour. The primary influence over the oral health behaviours of adolescents was parental. With maturity, the external environment (availability of sugar and peer influence) had an increasing impact on behaviour. Policy should be directed towards creating whole-life supportive environments conducive to support risk-minimising behaviours e.g. early-life support for parents and public policy on sugar availability.

## Abbreviations

D_3_MFT: decayed, missing, filled index
ELMS: East Lancashire and Manchester Study
IQR: interquartile range
IMDQ: index material deprivation quintile
